# Analysis of the Structure and Dynamics of European Flight Networks

**DOI:** 10.3390/e24020248

**Published:** 2022-02-08

**Authors:** Matteo Milazzo, Federico Musciotto, Salvatore Miccichè, Rosario N. Mantegna

**Affiliations:** 1 Dipartimento di Fisica e Astronomia Ettore Majorana, Università degli Studi di Catania, Via S. Sofia 64, I-95123 Catania, Italy; matteo.milazzo@dfa.unict.it; 2 Dipartimento di Fisica e Chimica Emilio Segrè, Università degli Studi di Palermo, Viale delle Scienze, Ed. 18, I-90128 Palermo, Italy; federico.musciotto@unipa.it (F.M.); rosario.mantegna@unipa.it (R.N.M.); 3 Complexity Science Hub Vienna, Josefstaedter Strasse 39, 1080 Vienna, Austria

**Keywords:** complex networks, network motifs, clustering, air transportation system

## Abstract

We analyze structure and dynamics of flight networks of 50 airlines active in the European airspace in 2017. Our analysis shows that the concentration of the degree of nodes of different flight networks of airlines is markedly heterogeneous among airlines reflecting heterogeneity of the airline business models. We obtain an unsupervised classification of airlines by performing a hierarchical clustering that uses a correlation coefficient computed between the average occurrence profiles of 4-motifs of airline networks as similarity measure. The hierarchical tree is highly informative with respect to properties of the different airlines (for example, the number of main hubs, airline participation to intercontinental flights, regional coverage, nature of commercial, cargo, leisure or rental airline). The 4-motif patterns are therefore distinctive of each airline and reflect information about the main determinants of different airlines. This information is different from what can be found looking at the overlap of directed links.

## 1. Introduction

The air transportation system (ATS) is a socio-technical system analyzed as a complex network for many years [1,2]. The ATS is analyzed at different geographical scales (see, for example, studies covering the ATSs of China [3], Europe [4] and the U.S. [5]) and at different resolutions starting from the airport–flight network down to the network of the reference points used in the definition of flight routes (called navigation points) [6].

In the majority of studies, the ATS is investigated by setting up a flight network where nodes are airports and flights connecting airports are links. The flight networks have been investigated by considering them undirected and/or directed networks (in this last case, the direction of the links originates from the departing airport and ends up in the arrival airport), unweighted and/or weighted [7]. Several studies have considered the problem of the resilience of the ATS to failures and attacks [5,8,9,10]. Other studies have selected a subset of links (labeled as the “backbone” of the ATS) presenting statistical properties that are not consistent with a specific null hypothesis [11,12], making the ATS one of the first systems where statistically validated networks [13] have been investigated.

The ATS is a complex system composed of well-defined subunits. In fact, flights are operated by different airlines that compete and collaborate among them. Since 2010, the ATS has been analyzed by distinguishing the role of its subunits (i.e., by analyzing properties of flight networks of single airlines [5]). Moreover, the presence of different flight networks observed for different airlines made this system a natural candidate for the study of so-called multiplex, which are networks where nodes can have multiple kinds of relations called layers. In fact, the ATS was one of the first socio-technical systems described as a multiplex, where layers represent flights operated by different airlines [14,15].

Flight networks have been investigated from different perspectives and at different scales [16,17,18], for example, by considering basic network metrics, topology of the degree distribution, resilience to attack or failures, community detection of large clusters and computation and analysis of network motifs. Motifs are isomorphic subnetworks of a specified number of nodes and shape. Motifs were first investigated in studies of social networks [19]. In these earlier studies, motifs were primarily investigated as triads (i.e., as subnetworks of three nodes) and put in relation with the properties of the degree sequence. At the beginning of this century, such structures were also investigated in biological systems under the name of motifs [20]. By considering isomorphic motifs (i.e., subnetworks where the identity of the node is not taken into account when considering the shape of the subnetwork), there are 13 isomorphic for subnetworks with 3 nodes or 3-motifs. This number soon explodes when the subnetwork includes more nodes. For subnetworks with 4 nodes (or 4-motifs), one counts 199 isomorphic motifs [20].

Network motifs have been investigated in flight networks both in studies comparing the informativeness of network detection in several types of complex networks [21] and in studies fully focused on the static and dynamics characteristics of the flight networks [22,23,24,25]. In this study, we investigate the temporal evolution of 3-motifs and 4-motifs for the 50 European airlines with the highest number of flights in the European Civil Aviation Conference (ECAC) airspace in the year 2017. By investigating the number and temporal evolution of the 3- and 4-motifs, we are able to perform an unsupervised classification of the 50 airlines indicating that main differences among different airlines are due to their regional specialization (including the ability to perform intercontinental flights) and to their business model. We observe that the business model of each airline ranges between the two stylized models of *hub-and-spoke* and *point-to-point* business models [26,27]. In a *hub-and-spoke* model, one or more airports act as “hubs”, i.e., as special airports directly connecting all remaining airports. In a *hub-and-spoke* structure with a single hub, the network therefore has a star topology with the hub at the center of the star and all the other airports acting as leaves of the network. In the *point-to-point* structure, all the airports are equivalent and the network degree is characterized by pair interconnections between airports.

The main goal of our investigation is a reliable and effective classification of airlines. The classification is obtained by an unsupervised methodology that only takes into account the information about the airline flights. We hypothesize that the business models of each airline induce specific constraints on its flight network. These constraints are reflected in the motif occurrence of each airline. Our network analysis shows that European airlines present a heterogeneous profile distributed between the two boundaries of *hub-and-spoke* and *point-to-point* business models. The heterogeneity is clearly shown by using a measure of concentration of degree in the degree sequence. Specifically, as a measure of concentration, we use an adapted version of the Herfindal–Hirshman index [28,29]. For the sake of simplicity, in the remaining text, we will call this index by the more traditional, although imprecise, name of Herfindal index.The time evolution of motifs shows that the basic temporal unit of the flight schedule is the week. Differences in the degree concentration observed during winter and summer schedules are detected, but their amount is negligible for most airlines. Average values of the motif occurrences may therefore be a useful proxy of the average behavior of the airlines over a calendar year. By using average values of the 4-motifs occurrence, we are able to obtain an unsupervised classification of airlines. The obtained hierarchical clustering is showing that the presence of a given number of hubs together with the presence or absence of intercontinental flights characterizes groups of airlines. On the other hand, a hierarchical clustering based on a similarity measure estimated starting from the co-presence of the two airlines in the origin–destination flight is providing a poorly informative hierarchical clustering.

The paper is organized as follows. In Section 2, we discuss the data used in our analysis and the metrics and methods used to characterize flight networks. In Section 3, we present our results about the heterogeneity of the degree concentration and our results about the structure and time evolution of 3- and 4-motifs for the different airlines. Average 4-motif occurrences are used to perform an unsupervised clustering of the 50 airlines providing an informative hierarchical cluster. In Section 4, we discuss our results.

## 2. Data and Methods

We investigate the flight networks of the 50 biggest commercial airlines flying over the European flight zone. Specifically, we consider all flights that occurred during the period from 1 January 2017 to 31 December 2017.

A flight network is a network where nodes are airports and links are flights that occurred in a given time interval. By considering that the flight occurs from a departing airport to an arrival airport, flight networks can be described as directed weighted networks (where the weight of a link is the number of flights that occurred from airport *i* to airport *j* in the chosen time interval). In this study, we considered flight networks as directed networks while we disregard the weights of the links. Networks are computed using daily and weekly time intervals.

Flight networks and their metrics of each airline are analyzed both in their time evolution and in their subunits. Specifically, we investigate the daily degree sequence of each airline for each day. In our analysis, we primarily focus on the concentration of the highest degree values on a limited set of airports usually described as “hubs”. This is performed by adapting the Herfindal index, i.e., a well-known measure of concentration, to the degree sequence. The subunits analysis is carried out by considering all isomorphic small networks with 3 or 4 nodes. These subnetworks are called motifs in the biological literature or triads or subnetworks in the social science literature.

We compare similarity between pairs of airlines both by considering the links, i.e., flights, they are performing on a specific day or week and by considering the motifs they present on a specific day or on average over the full year. Similarity between the airlines is therefore estimated and interpreted by extracting hierarchical trees from the selected similarity matrix.

### 2.1. Flight Data

Our dataset comprises all the flights that, even partly, cross the ECAC airspace for the entire 2017 year. Data were obtained by EUROCONTROL (http://www.eurocontrol.int, accessed on 4 February 2022), the European public institution that coordinates and plans air traffic control for all of Europe.

Specifically, we obtained access to the Demand Data Repository (DDR) from which one can obtain all flights followed by any aircraft in the ECAC airspace. Data about flights contain several types of information. In the present study, we just focus on the origin–destination of each flight crossing the ECAC airspace at a given time.

By considering that our focus is on the specific characteristics of airlines, in the present study, we investigate flights of the major 50 airlines performing flights in the ECAC airspace in 2017. In our set, we do not consider Air Berlin because this airline ceased operations on 27 October 2017. Since 2016, Germanwings has been a lease operator for its sister company Eurowings. In our set, we are not considering Germanwings flights. The selected airlines have performed 65.7% of the total number of flights of 2017, which corresponds to approximately 3000 flights per company per month on average. The list of the 50 airlines is provided in Appendix A. The large majority of airlines are commercial airlines. There are 24 flag carrier airlines, 14 low cost carrier (LCC) airlines, 6 regional airlines, 2 leisure airlines, 2 scheduled airlines, 1 cargo airline and 1 rental airline.

### 2.2. Herfindal Index

The Herfindal index [28] has been introduced in the economic literature in order to measure the amount of competition among industrial firms. As such, it has also been used as an indicator of concentration, as large firms usually contribute more to the Herfindal index than smaller ones. In the context of complex networks, the Herfindal index can be defined as

(1) $$H\;=\;\sum_{i\;=\;1}^{N}{\left[\frac{d_{i}}{2m}\right]}^{2}$$

where $d_{i}$ is the degree of node *i* and 2m is twice the number of directed links.

### 2.3. Motifs Detection

The investigation of subnetworks of fixed size (also called motifs) has a long history. Originally investigated as triads and put in relation with the properties of the degree sequence in the investigation of social networks [19], they were then also introduced in biology where the term “motif” was used for the first time [20].

**Figure 1:**
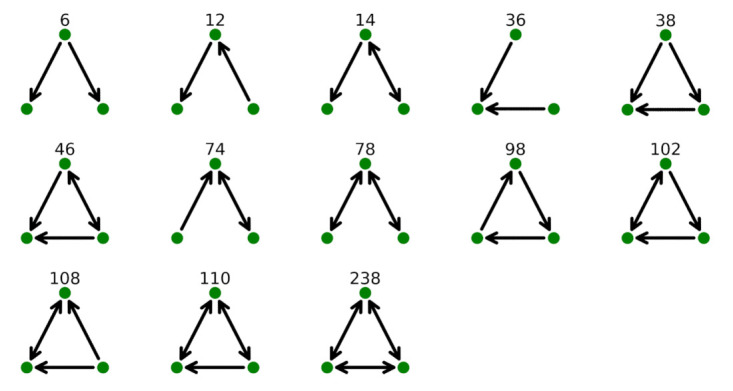
Isomorphic 3-motifs and related codes according to the classification given in [20]. The arrow indicates a flight from origin (tail) to destination (head). Bidirectional flights are indicated with a double head arrow.

In network analysis, a motif of size k is a structure of k nodes not necessarily all linked between each other, as, for example, in Figure 1. Motifs are different from cliques. A clique is defined in undirected networks, and it is a subgraph such that every two distinct vertices are adjacent.

For size k = 3, there are 13 isomorphic 3-motifs. In Figure 1, we are showing all of them together with the classification scheme used in [20]:

Isomorphic 3-motifs present unidirectional links (as in the case of motifs labeled as 6, 12, 36, 38 and 98), bidirectional links (as in the case of motifs 78 and 238) and both types of links (as in the case of motifs 14, 46, 74, 102, 108 and 110).

The number of isomorphic 4-motifs is 199 and therefore much larger than 13. As for the 3-motifs, we use the classification of [20]. For the shape of each 4-motif, one can consult the motifs dictionary that can be downloaded from the website of Uri Alon laboratory.

Network motif analysis can be performed by computational or analytical approaches. In our investigation, we considered a computational approach as it allows for the exact count of network motifs. Computational approaches usually follow a three-step procedure that can be summarized as follows:

- Search and enumerate occurrences of a topology with fixed size in the observed network;
- Classify topologies by their isomorphic classes;
- Calculate statistical significance for each isomorphic classes comparing occurrences with those in random ensemble.

In particular, we considered the *mfinder* [30] software developed by Uri Alon laboratory.

### 2.4. Average Linkage Clustering Analysis

We assess the similarity between each pair of the *n* airline by estimating the correlation between the average occurrence of each 4-motif of each airline. The average is computed over the 365 days of the year. To take into account the large interval of values observed for the different 4-motifs, we use the Spearman correlation coefficient. Therefore, by starting from the matrix of records obtained by averaging the occurrence of each 4-motif, we estimate a correlation matrix and we use the correlation $\rho_{ij}$ as a measure of similarity between airlines *i* and *j*.

From the correlation values, we compute a distance according to the relation $d_{ij}\;=\;\sqrt{2(1\;-\;\rho_{ij})}$. This distance is therefore used to extract a hierarchical tree with the method of the average linkage.

The average linkage cluster analysis is a hierarchical clustering procedure [31,32]. The procedure gives as an output a rooted tree or dendrogram. In this procedure, at each step, when two elements or one element and a cluster or two clusters *p* and *q* merge in a wider single cluster *t*, the distance $d_{tr}$ between the new cluster *t* and any cluster *r* is recursively determined as the average distance between any element of *t* and any other element of cluster *r*.

## 3. Results

### 3.1. Herfindal Index

**Figure 2:**
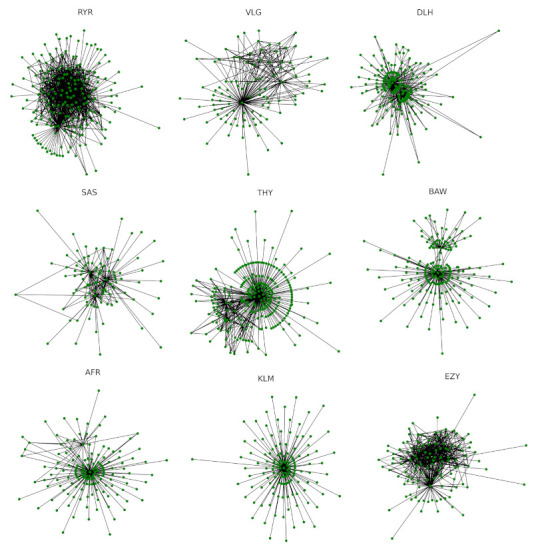
Airline networks on day 1 September 2017. Each panel refers to one of the nine biggest airlines. The airline name is indicated on top of the panel.

Our first analysis determines the daily flight network of each investigated airline. The day is defined as the calendar day at European Central Time. For illustrative purposes, we show the networks of the nine biggest airlines on day 1 September 2017 in Figure 2. This day has been retrospectively selected as an example of a day with routinely operational activities.

For each flight network, we extract the degree sequence by considering the network as a directed network. The average values over the year of the number of nodes *N* (i.e., number of airports where airlines flight), the number of direct links *E* (i.e., the number of distinct origin destination flights), minimum degree, median degree, mean degree, maximum degree, standard deviation of the degree and Herfindal index are shown in Table 1. The metrics shown in Table 1 are quite basic and standard with the exception of the adaptation of the Herfindal index as an indicator of concentration in the degree sequence observed in one or more of the nodes.

**Figure 3:**
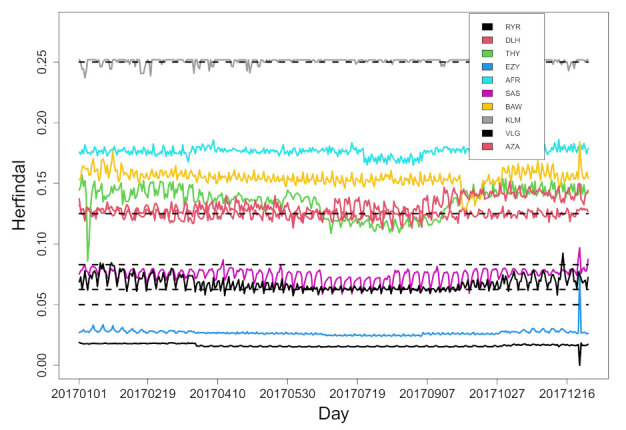
Herfindal index of flight networks (defined as in Equation (1)) as a function of the calendar day for the top 10 airlines Ryanair (RYR), Lufthansa (DLH), Turkish Airlines (THY), EasyJet (EZY) Air France (AFR), Scandinavian Airlines (SAS), British Airways (BAW), KLM (KLM), Vueling Airlines (VLG) and Alitalia (AZA).

Given the definition of Equation (1), a pure *hub-and-spoke* setting of flights would imply a Herfindal index of 0.25 for large values of *N*. This is what we observe (see Table 1) as average yearly value for Brussels Airlines (BEL), Aeroflot (AFL), KLM, Iberia Airlines (IBE) and Finnair (FIN). Networks of these airlines are very close to a pure *hub-and-spoke* setting. Other airlines show lower values of the average Herfindal index. The values observed range from 0.213 for Austrian Airlines to 0.016 for Ryanair, showing a high variability of the underlying flight network structure. For the sake of compactness, in Table 1, we show only the yearly average values of the selected indicators. To assess the degree of variability of the Herfindal index, we show in Figure 3 the daily profile of this index for the top ten airlines in number of flights. They are Ryanair (RYR), Lufthansa (DLH), Turkish Airlines (THY), EasyJet (EZY) Air France (AFR), Scandinavian Airlines (SAS), British Airways (BAW), KLM (KLM), Vueling Airlines (VLG) and Alitalia (AZA). Time dynamics of the Herfindal index is detectable for several airlines but fluctuations are quite limited and primarily reflect a weekly or intra-weekly periodicity. Some airlines also show detectable winter–summer dynamics. Examples are THY, AZA and VLG. Horizontal dashed line is the expected values of the Herfindal index for networks with only bidirectional links and with *K* pure hubs and all the remaining (large) number of leaves only flying to a single hub for *K* ranging from one (top dashed line) to five (bottom dashed line). In particular, KLM networks are compatible with a network structure having a single hub (i.e., Schipol airport), Lufthansa (DLH) networks are compatible with a two hub network (prominent Lufthansa hubs are Frankfurt and Munich airports). Vueling (VLG) and Scandinavian Airlines (SAS) have a pattern compatible with three or more hubs and/or with a prominent section of the flight network based on *point-to-point* flight circulation, whereas the Herfindal index of Ryanair and EasyJet have pretty low values, manifesting the poor relevance of the *hub-and-spoke* structure in their flight networks.

Our analysis can therefore confirm that flight network characteristics are deeply related to the business organization of each airline with a prominent role played by the choice of a *hub-and-spoke* versus a *point-to-point* structure and with a role played by the number of hubs characterizing the flight network.

In the next section, we investigate 3-motifs to better characterize similarity and differences among the flight networks of airlines.

### 3.2. 3-Motifs

**Figure 4:**
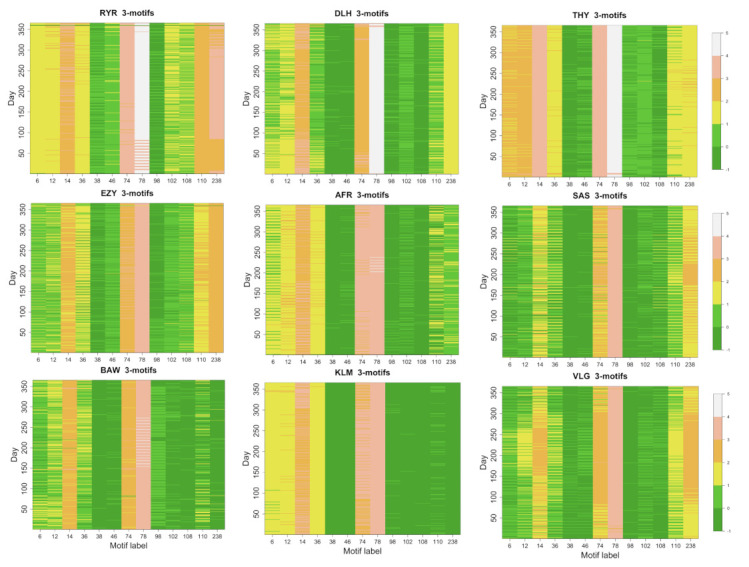
Color code representation of the base 10 logarithm of the daily occurrence of each isomorphic 3-motif (*x*-axis) as a function of the time (*y*-axis). The 3-motifs are indicated with the labels defined in [20]. A zero occurrence is indicated with a −1 value. Each panel refers to one of the nine biggest airlines. The airline name is indicated on top of the panel.

We have computed the number of 3-motifs present on daily flight networks for all 50 airlines. In Figure 4, we show a color code map of the occurrence of the 13 isomorphic 3-motifs for the 9 largest airlines.

The occurrence of each 3-motif presents large variability among the different types of motifs and is correlated with properties of the flight networks such as number of nodes, number of links, number of bidirectional links and topology structure of the network. The most common 3-motif is motif 78. This type of motif is clearly manifesting that a hub-like structure and bidirectional links are essential ingredients of all flight networks. The other 3-motif with all bidirectional link, i.e., 3-motif 238, is significantly present in airlines presenting flight networks with a pronounced *point-to-point* structure, such as Ryanair, EasyJet and Vueling, or airlines having more than a single hub, such as Lufthansa, Turkish and Scandinavian Airlines. The 3-motifs with only unidirectional links are poorly observed (see average occurrence values of 3-motifs 6, 12, 36, 38 and 98). Some of the 3-motifs with mixed types of links are significantly present (for example, 3-motifs 14 and 74), while others are rather poorly expressed (as in the case of 3-motifs 102 and 108).

The profile of occurrence of the 3-motifs in different airlines is certainly informative. However, the number of 3-motifs is somewhat limited and therefore it is useful to consider motifs of larger size. In the next section, we investigate the occurrence of 4-motifs.

### 3.3. 4-Motifs

#### 3.3.1. Daily Occurrence of 4-Motifs

**Figure 5:**
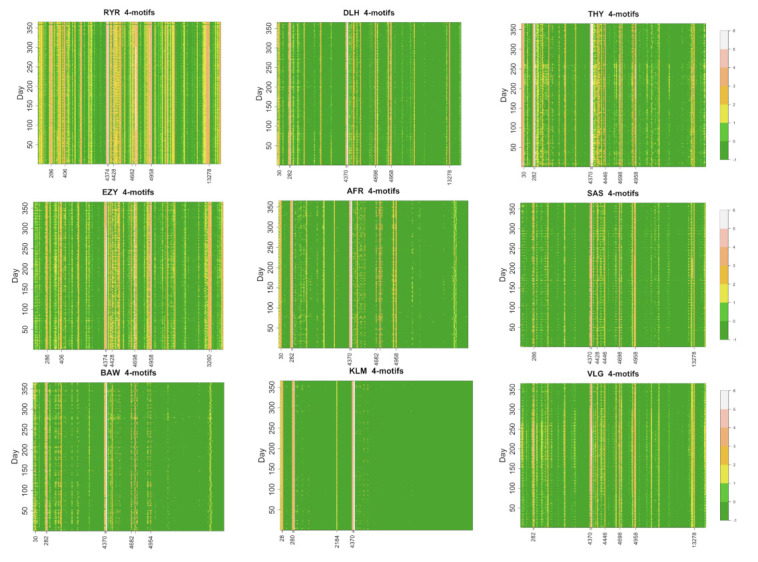
Color code representation of the base 10 logarithm of the daily occurrence of each isomorphic 4-motif (*x*-axis) as a function of the time (*y*-axis). The 4-motifs are ranked according to the labels defined in [20]. Some of the most populated 4-motifs are indicated in the *x*-axis. A zero occurrence is indicated with a −1 value. Each panel refers to one of the nine biggest airlines. The airline name is indicated on top of the panel.

We compute the occurrence of all 4-motifs for the daily flight networks of the 50 biggest airlines. In Figure 5, we show a color code map of the occurrence of the 199 isomorphic 4-motifs for the 9 largest airlines.

**Figure 6:**
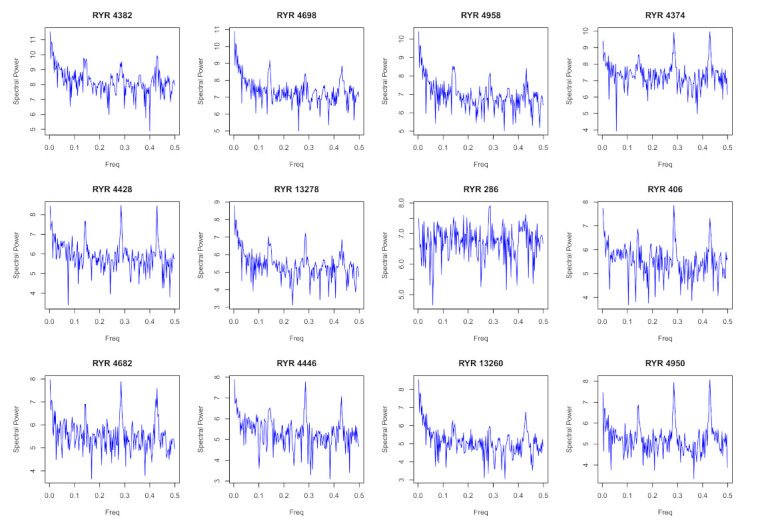
Power spectrum of the time evolution of the occurrence of the top 12 4-motifs of Ryanair. The label of the 4-motif is on top of each panel. Frequency peaks are detected for f=0.14 and for its harmonics f=0.28 and *f* = 0.43 day${}^{-1}$. The main frequency *f* = 0.14 day${}^{-1}$ corresponds to a weekly cycle and the second and third harmonics correspond to a bi-weekly or three-weekly cycle.

The profile of 4-motifs is richer than the one of the 3-motifs. Occurrences of the 4-motifs span about 5 orders of magnitude. For this reason, in Figure 5, we show the decimal logarithm to provide a comprehensive overview of the results. Airlines characterized by the presence of a single hub such as KLM present only a very limited number of 4-motifs with occurrence different from zero. Airlines with a business model closer to a *point-to-point* structure such as Ryanair and EasyJet present a higher number of observed 4-motifs. The other airlines characterized by a different number of hubs present an intermediate behavior between the two extremes. In addition to the presence or absence of a given motif at a given day, Figure 5 also shows a time variation of the occurrence of a given motif. To investigate the main frequencies associated with this time variation, we compute the periodogram of the occurrence of a set of 4-motifs. Specifically, we consider the twelve 4-motifs with the highest occurrence averaged over all considered days. In Figure 6, we show the power spectrum of the time evolution of the occurrence of the top twelve 4-motifs of Ryanair. For all 4-motifs, frequency peaks are detected for *f* = 0.14 day${}^{-1}$ and for its second and third harmonics. The main frequency *f* = 0.14 day${}^{-1}$ corresponds to a weekly cycle and the second and third harmonics correspond to a bi-weekly or three-weekly cycle. Therefore, the main underlying periodicity is the week periodicity as already observed in the estimation of the Herfindal index (see periodicity observed in Figure 3).

Tracking in details of the occurrence for the 199 different 4-motifs is impractical. For this reason, we first consider the ten 4-motifs with highest occurrence in the nine biggest airlines. Specifically, we rank these 4-motifs, and the rank is obtained by considering the daily occurrence of each motif averaged over all days of the year. Labels of these motifs are listed on Table 2 according to their rank for the 9 biggest airlines.

**Figure 7:**
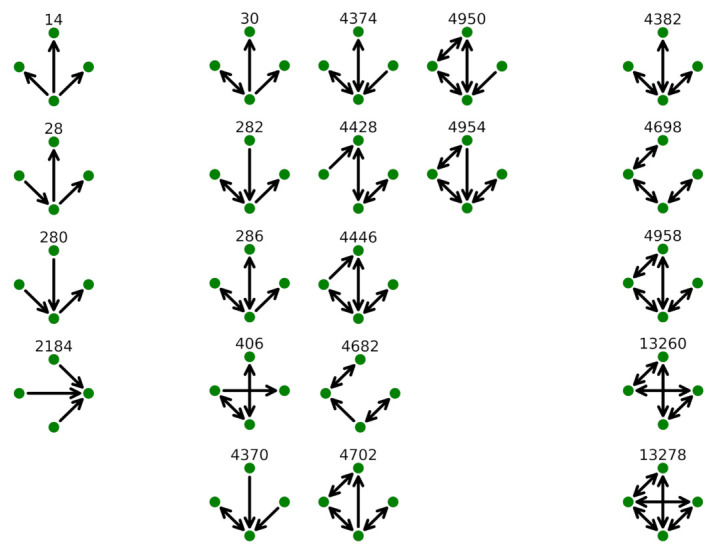
Shape of the 10 top 4-motifs observed for the 9 largest airlines (see Table 2). The 4-motifs are labeled according to [20]. Left column groups 4-motifs with only unidirectional links. Right column groups 4-motifs with only bidirectional links. Center column groups 4-motifs with both unidirectional and bidirectional links.

The link configuration of these 4-motifs is shown in Figure 7. In the figure, we show on the left 4-motifs composed by unidirectional links, whereas on the right we have motifs with only bidirectional links. The 4-motifs with both unidirectional and bidirectional links are shown in the middle of the figure. It is worth noting that 4-motifs with only unidirectional links (i.e., 4-motifs 14, 28, 280 and 2184) are only observed for KLM in the top 10. KLM is one of the airlines with an almost pure *hub-and-spoke* structure and the flight concurring to this type of motif is, in the majority of the cases, an intercontinental flight. All the other airlines have the top 10 4-motifs presenting a high number of bidirectional links. The 4-motif with the highest occurrence for all the top 9 airlines (and indeed the top 4-motif for 49 of 50 airlines) is 4-motif 4382. These motifs present three bidirectional links originating from the same node. As for 3-motif 78, the largest occurrence of this motif reflects the fact that at least one important airport is used as a hub by the airline generating the network. The other 4-motifs composed by only bidirectional links (i.e., 4-motifs 4698, 4958, 13260 and 13278) are compatible with a *point-to-point* structure or with a *hub-and-spoke* structure in the presence of at least two hubs. In fact, these 4-motifs are not observed for KLM and are observed at the highest rank for more oriented *point-to-point* airlines such as Ryanair, EasyJet and Vueling. They are also present when more than one hub is present as, for example, in the case of Lufthansa or Scandinavian Airlines. The ranking of the 4-motifs can therefore be used to evaluate the similarity of flight airline networks and we investigate this possibility in the next section.

#### 3.3.2. Similarity of 4-Motif Profile

We use the information about the 4-motifs occurrence to obtain a categorization of airlines by using the methodology of Section 2.4. It is worth recalling here that, given this specific purpose, it is not necessary for us to maintain the information about the specific airports that is present in a motif. In fact, since we are interested in extracting a clusterization of airlines by using the structural information about the 4-motifs, only the isomorphic motifs will be relevant for us.

**Figure 8:**
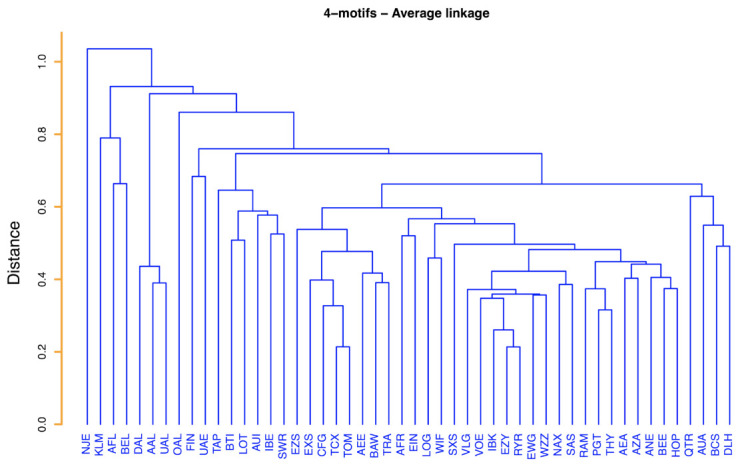
Average linkage hierarchical tree obtained by using the relation $d_{ij}=\sqrt{2(1-\rho_{ij})}$ as distance, where $\rho_{ij}$ is the Spearman correlation coefficient between the vectors of the average occurrence of each 4-motif of airlines *i* and *j*. The average is computed over the 365 days of the year.

The result of our analysis is shown in Figure 8.

The hierarchical tree of Figure 8 is highly informative with respect to the clustering of groups of airlines. One airline markedly distinct from all others is NetJets Transportes Aéreos, S.A. (NJE). This airline is the only airline of the set providing rental of jets and therefore observing it distinct from all the others indicates that the observed flights of this rental company have 4-motifs that are quite distinct from the ones of all other airlines. An inspection of the hierarchical tree indicates the presence of clusters of airlines presenting a certain similarity among them and a degree of dissimilarity from the other airlines. Here, we wish to comment about some of them. One cluster is the cluster of KLM, Aeroflot (AFL) and Brussels Airlines (BEL). These three airlines are airlines with a single large hub as testified by a Herfindal index very close to 0.25 (see Table 1). Another cluster comprises Delta Air Lines (DAL), American Airlines (AAL) and United Airlines (UAL). These three airlines are American airlines primarily performing intercontinental flights. A large cluster is composed by Vueling Airlines (VLG), Volotea (VOE), Norwegian Air International (IBK), EasyJet (EZY), Ryanair (RYR), Eurowings (EWG), Wizz Air (WZZ), Norwegian Air Shuttle (NAX) and Scandinavian Airlines (SAS). These are all airlines with several hubs and/or with a *point-to-point* business model. Another cluster comprises Royal Air Maroc (RAM), Pegasus (PGT) and Turkish Airlines (THY). These airlines are primarily serving Middle East destinations and airlines are headquartered in Middle East countries. Another distinct cluster comprises Qatar Airways Company Q.C.S.C. (QTR), Austrian Airlines (AUA), European Air Transport Leipzig (BCS) and Lufthansa (DLH). With the exception of Qatar Airways, the airlines of this cluster are all based in central Europe. In fact, Lufthansa and European Air Transport Leipzig are German airlines (Lufthansa is the second largest commercial airline in Europe and European Air Transport Leipzig is the largest cargo company in Europe by number of flights) and Austrian Airlines is a subsidiary of the Lufthansa Group.

### 3.4. Airline Networks Overlap

It is worth estimating whether similarity between 4-motif occurrences could just be due to overlap between the links of the flight network of airlines. We rule out, to a large extent, this possibility by investigating the degree of overlap between all pairs of airline networks. Our investigation is conducted by estimating the Jaccard measure $J(net_{1},net_{2})$ between each pair of airline networks $net_{1}$ and $net_{2}$. The Jaccard similarity is defined as

(2) $$J\left(net_{1},net_{2}\right)\;=\;\frac{|E_{1}\cap E_{2}|}{|E_{1}\cup E_{2}|},$$

where $|E_{1}\cap E_{2}|$ is the number of directed links appearing in both flight networks, and $|E_{1}\cup E_{2}|$ is the number of links that appear in at least one of the two networks.

**Figure 9:**
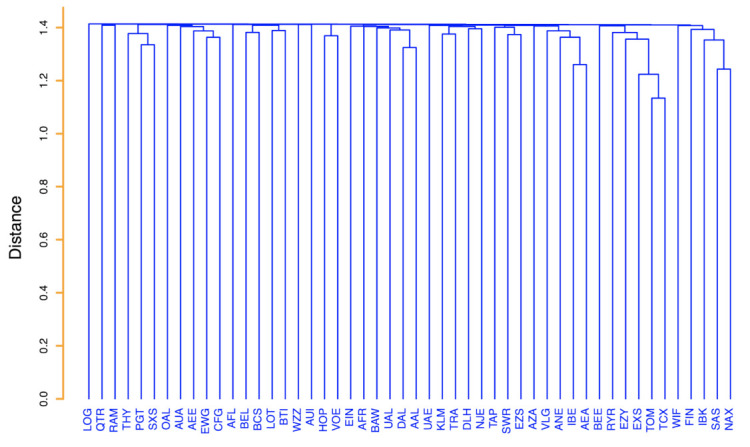
Average linkage hierarchical tree obtained by using as distance the relation $d_{ij}=\sqrt{2(1-J\left(net_{i},net_{j}\right))}$, where $J(net_{i},net_{j})$ is the Jaccard measure of the overlap of directed links observed in weekly flight network *i* and *j*. The hierarchical tree is obtained for the 20th week of 2017 (from Monday 15 May to Sunday 21 May).

To take into account weekly variability of flight schedules, we have performed this analysis by considering the weekly schedule of each airline. The results obtained at the daily level are showing a degree of similarity of the same order or less. In Figure 9, we show the average linkage hierarchical tree obtained by using the Jaccard measure as a similarity measure. The hierarchical tree is poorly informative and only a very limited number of small clusters can be highlighted. This is in marked contrast with what we have obtained in the previous section when the similarity measure between airlines was obtained from the analysis of 4-motifs. The hierarchical tree shown in Figure 9 is representative of hierarchical trees obtained for all weeks of 2017.

By summarizing, we are the first to use the Herfindal index to characterize each airline operating in a given period. Moreover, by using the Herfindal index together with 3- and 4-motif analysis, we are able to achieve an unsupervised classification of airlines, clarifying the main characteristics of each airline.

## 4. Discussion and Conclusions

In the present study, we have analyzed the structure and dynamics of flight networks of 50 airlines performing most of the flights that occurred in the European airspace in 2017. Our analysis of directed flight networks shows that the degree concentration of the different networks is quite heterogeneous among the different airlines. We have been able to quantify this heterogeneity by using an adapted version of a classic measure of concentration, i.e., the Herfindal index. The Herfindal index provides a simple and reliable estimation of the closeness of the airline network to the reference models classified as *hub-and-spoke* and *point-to-point*. It can also be informative about the number of main hubs that are present in a network with a *hub-and-spoke* structure and multiple hubs. It is worth noting that the European ATS presents a very heterogeneous set of airline companies. In other words, business optimization performed at the level of a single airline generates different business models that eventually coexist in the global system.

The time evolution of the different networks presents a basic time cycle that is a weekly cycle. This basic timescale is evident both from the analysis of the time evolution of the Herfindal index and from the analysis of the time evolution of the occurrence of 3-motifs and 4-motifs. The summer–winter cycle primarily detected in the number of flights occurring daily or weekly does not significantly affect the long-term time evolution of the Herfindal index and the occurrence of 3-motifs and 4-motifs. These indicators are therefore more related to the type of business model followed by the airline than to the specific origin–destination links or number of flights operated in a given time interval.

In summary, an unsupervised classification based on hierarchical clustering and obtained by using a correlation coefficient between the occurrence profile of 4-motifs of airline networks as similarity measure is highly informative with respect to the properties of the different airlines (for example, the number of main hubs, their participation to intercontinental flights, their regional coverage, their nature of commercial, cargo, leisure or rental airline). The 4-motifs are therefore distinctive of the airlines and reflect information about the main determinants of the different airlines. Information is distinct from that originating from the overlap of the same directed links.

Such results indicate that a reliable and effective classification of airlines can be obtained by an unsupervised methodology that only takes into account the information about the airline flights. This is an important result given that, currently, the characterization of airlines and their business model has become a fundamental part of modern air transportation systems. An appropriate airline categorization is important not only for the practitioners but because it also influences the passengers’ perception. An indubitable advantage of our approach is that it is flexible as it may directly reflect any positioning of an airline within the general landscape of airlines, due to any change in its business model as reflected within its flight plans.

## Figures and Tables

**Table 1:** Basic metrics of the flight networks of 50 largest airlines by number of flights performed. The degree of each airport is obtained as the average value of the degree observed for each day of the year. N is the number of nodes, *m* is the number of direct links, Min, Median, Mean, Max and Std are the minimum, median, mean, maximum and standard deviation of the average degree distribution. The Herfindal index is obtained from the average degree distribution as explained in the main text. Airlines are ordered according to the value of the Herfindal index.

Airline	N	2m	Min	Median	Mean	Max	Std	Herfindal
BEL	65.08	239.65	1.00	2.00	3.68	119.52	14.57	0.253
AFL	63.87	248.19	1.17	2.00	3.89	123.30	15.18	0.251
KLM	143.69	538.27	1.00	2.00	3.75	268.50	22.24	0.251
IBE	60.39	227.55	1.00	2.00	3.77	110.84	14.01	0.243
FIN	78.79	308.37	1.00	2.00	3.92	147.95	16.43	0.234
AUA	82.96	342.75	1.01	2.00	4.13	155.99	16.90	0.213
TAP	76.26	327.58	1.00	2.00	4.30	144.44	16.40	0.202
QTR	67.19	266.83	1.00	2.00	3.97	117.99	14.15	0.201
BTI	36.78	160.37	1.06	2.00	4.37	67.62	10.84	0.192
SWR	78.68	351.81	1.00	2.00	4.47	148.66	16.76	0.190
AUI	54.36	233.90	1.01	2.00	4.29	98.64	13.13	0.189
UAE	63.07	247.10	1.00	2.00	3.92	103.43	12.80	0.183
LOT	66.62	288.13	1.03	2.00	4.32	119.29	14.47	0.182
AFR	156.12	657.34	1.00	2.00	4.21	270.10	21.76	0.176
OAL	26.62	125.88	1.89	2.00	4.72	48.19	9.04	0.174
RAM	79.28	327.75	1.00	2.00	4.13	132.78	14.75	0.172
EIN	52.90	238.28	1.07	2.00	4.48	92.72	12.46	0.164
BAW	162.38	660.51	1.00	2.00	4.07	244.37	20.01	0.155
AEE	50.90	236.93	1.01	2.00	4.59	79.58	11.08	0.141
PGT	89.83	459.42	1.01	2.00	5.10	160.00	17.08	0.138
THY	245.36	1274.18	1.00	2.00	5.18	449.51	29.37	0.135
EZS	42.87	221.23	1.08	2.20	5.15	61.88	11.28	0.133
AZA	77.52	410.59	1.01	2.00	5.29	138.81	16.15	0.132
AEA	50.64	237.18	1.01	2.00	4.69	77.37	10.93	0.126
DLH	175.87	1015.78	1.00	2.27	5.77	289.74	26.45	0.124
SXS	33.31	199.47	1.04	3.58	5.91	46.17	9.71	0.113
ANE	38.43	198.79	1.27	2.17	5.13	54.17	8.82	0.104
TRA	54.48	286.73	1.01	2.07	5.24	75.58	10.87	0.098
UAL	31.36	201.63	1.16	3.47	6.39	47.35	8.79	0.091
LOG	19.34	109.38	1.62	3.33	5.62	19.33	4.90	0.089
BCS	59.85	288.98	1.23	1.86	4.62	60.62	8.46	0.080
NAX	67.06	401.90	1.02	2.21	5.99	87.38	12.48	0.079
HOP	35.22	214.97	1.06	2.18	6.08	40.50	7.84	0.076
DAL	35.56	210.47	1.09	2.73	5.87	36.18	7.61	0.075
SAS	94.26	652.47	1.09	2.62	6.89	110.63	16.92	0.074
AAL	23.42	157.98	1.55	4.86	6.61	22.76	5.69	0.074
CFG	41.51	234.10	1.00	2.20	5.53	44.99	7.83	0.074
TCX	33.97	222.88	1.00	3.56	6.09	33.87	6.82	0.070
VLG	86.65	689.10	1.07	3.60	7.90	153.70	17.31	0.067
EWG	71.22	465.41	1.00	2.48	6.38	73.06	11.45	0.062
EXS	34.71	332.78	1.16	5.87	8.82	35.44	8.22	0.059
VOE	35.79	230.88	1.14	4.21	5.84	21.07	4.78	0.054
IBK	51.23	381.92	1.00	3.32	7.46	43.99	9.31	0.050
TOM	46.92	370.30	1.00	4.55	7.59	40.19	8.45	0.050
BEE	46.38	429.76	1.31	5.35	9.24	43.37	9.74	0.045
WIF	43.98	325.59	1.69	5.57	7.29	25.35	5.85	0.039
EZY	110.22	1714.64	1.08	7.32	15.44	157.97	21.53	0.027
WZZ	100.23	895.02	1.00	5.33	8.87	60.08	11.02	0.026
NJE	90.32	262.93	1.00	1.98	2.88	16.03	2.77	0.022
RYR	172.47	3183.89	1.00	9.64	18.33	206.09	24.72	0.016

**Table 2:** Rank of the 10 top 4-motifs observed for the 9 biggest airlines. The rank is obtained by considering the daily occurrence of each motif averaged over all days of the year. The shape of these 4-motifs are shown in Figure 7.

Rank	RYR	DLH	THY	EZY	AFR	SAS	BAW	KLM	VLG
1	4382	4382	4382	4382	4382	4382	4382	4382	4382
2	4698	286	286	4698	4374	4374	286	4374	4374
3	4958	4374	4374	4958	286	4958	4374	286	4958
4	4374	4958	282	4374	282	4698	4682	282	286
5	4428	13,278	30	13,278	4370	286	282	4370	4698
6	13,278	282	4370	4428	30	13,278	4698	30	4446
7	286	30	4958	13,260	4698	4370	30	280	13,278
8	406	4698	4698	286	4958	4428	4370	28	282
9	4682	4370	4446	406	4682	4446	4954	2184	4370
10	4446	4446	28	4950	280	4950	4702	14	4950

## Data Availability

Restrictions apply to the availability of these data. Data were obtained from EUROCONTROL.

## Appendix A

The table below contains the list of airlines considered in this work and some information about their business model. Such data were obtained from the following links (accessed on 4 February 2022):

- https://en.wikipedia.org/wiki/List_of_low-cost_airlines;
- https://en.wikipedia.org/wiki/List_of_regional_airlines;
- https://en.wikipedia.org/wiki/List_of_government-owned_airlines;
- https://en.wikipedia.org/wiki/List_of_charter_airlines;
- https://www.eraa.org/membership/our-members;
- Wikipedia pages of airlines.

**Table A1:** Fifty airlines performing the largest number of flights in the ECAC airspace in 2017. Airlines are ordered by the total number of flights performed in 2017. The last column gives the number of flights performed by the airline in 2017.

Airline	ICAO Code	Type	# of Flights
Ryanair	RYR	LCC	815,878
Lufthansa	DLH	Flag	561,437
Turkish Airlines	THY	Flag	555,720
EasyJet	EZY	LCC	538,533
Air France	AFR	Flag	369,991
Scandinavian Airlines	SAS	Flag	330,515
British Airways	BAW	Flag	304,112
KLM	KLM	Flag	274,822
Vueling Airlines	VLG	LCC	219,839
Alitalia	AZA	Flag	215,033
Wizz Air	WZZ	LCC	186,713
Pegasus	PGT	LCC	179,942
Flybe	BEE	Regional	169,299
Swiss	SWR	Flag	156,173
TAP Portugal	TAP	Flag	148,363
Austrian Airlines	AUA	Flag	141,305
Norwegian Air Shuttle	NAX	LCC	138,010
Eurowings	EWG	LCC	133,738
Finnair	FIN	Flag	128,253
Wideroe	WIF	Regional	127,047
Aeroflot	AFL	Flag	122,480
LOT Polish Airlines	LOT	Flag	117,962
Iberia Airlines	IBE	Flag	105,055
Norwegian Air International	IBK	LCC	104,835
Qatar Airways Company Q.C.S.C.	QTR	Flag	99,298
Air Europa	AEA	Scheduled	92,521
Brussels Airlines	BEL	Flag	92,279
Emirates	UAE	Flag	90,894
Royal Air Maroc	RAM	Flag	90,626
Aer Lingus	EIN	Flag	87,330
Air Nostrum	ANE	Regional	82,254
Hop!	HOP	Regional	79,385
European Air Transport Leipzig	BCS	Cargo	76,697
TUI Airways	TOM	Leisure	74,834
Jet2.com	EXS	LCC	69,338
Transavia Holland	TRA	LCC	68,758
United Airlines	UAL	Flag	67,796
Ukraine International	AUI	Flag	64,963
Delta Air Lines	DAL	LCC	64,552
Aegean Airlines	AEE	Flag	59,762
Olympic Air S.A.	OAL	Regional	58,135
SunExpress TK	SXS	LCC	55,761
EasyJet Switzerland SA	EZS	LCC	54,120
Air Baltic	BTI	Flag	52,722
American Airlines	AAL	Flag	52,118
Volotea	VOE	LCC	49,722
NetJets Transportes Aereos, S.A.	NJE	Rental	49,338
Condor Flugdienst	CFG	Leisure	46,257
Logonair	LOG	Regional	45,688
Thomas Cook Airlines Limited	TCX	Scheduled	45,230
